# From Awareness to Adoption: Barriers and Facilitators of Telerehabilitation in Jordanian Physiotherapy Practice

**DOI:** 10.3390/healthcare14172685

**Published:** 2026-08-24

**Authors:** Sami Elmahgoub, Wesam A. Debes, Rama Al-rawajfeh, Fares G. Daradkeh, Bodor Bin Sheeha, Aseel Aburub

**Affiliations:** 1 Department of Physiotherapy, Faculty of Allied Medical Sciences, Applied Science Private University, Amman 11937, Jordan; w_aldebes@asu.edu.jo (W.A.D.); r_alrawajfeh@asu.edu.jo (R.A.-r.); 2023419@students.asu.edu.jo (F.G.D.); a_abualrob@asu.edu.jo (A.A.); 2 Awareness in Physiotherapy Research Group, Faculty of Allied Medical Sciences, Applied Science Private University, Amman 11937, Jordan; 3 Department of Rehabilitation Sciences, College of Health and Rehabilitation Sciences, Princess Nourah Bint Abdulrahman University, Riyadh 11671, Saudi Arabia; bhbinsheeha@pnu.edu.sa

**Keywords:** telerehabilitation, physiotherapy, Jordan, awareness, barriers, telehealth, digital health

## Abstract

**Background/Objectives:** Telerehabilitation is increasingly used globally to deliver physiotherapy services remotely, yet its adoption in Jordan remains limited. This quantitative cross-sectional study aimed to explore the awareness, perceptions, and willingness of Jordanian physiotherapists regarding telerehabilitation, and to identify barriers and facilitators to its adoption. **Methods:** An online self-administered questionnaire was completed by a convenience sample of 200 physiotherapists (111 males, 89 females; age range 23–62 years). Descriptive statistics and chi-square tests were used for analysis. **Results:** Results revealed that 116 participants (58.0% of the total sample) were aware of telerehabilitation. Among those aware, 79 (68.1%) had used it, with online video (27.0% of total sample; 46.6% of users) being the most common modality. A significant association was found between higher education level (Master’s/Ph.D.) and greater awareness (*p* < 0.001). Key barriers included lack of reimbursement (33.0% of total sample; 56.9% of aware respondents), technical difficulties (47.0%), infrastructure issues (39.0%), and lack of training (34.0%). Among aware participants who responded to attitude items, the majority agreed that telerehabilitation enhances patient access (85/116, 73.3% of aware respondents) and is a valuable addition to practice (102/116, 87.9% of aware respondents), while 43.0% of the total sample (86/200) believed it can never fully replace in-person consultation. **Conclusions:** Awareness of telerehabilitation among Jordanian physiotherapists is moderate, and actual clinical use remains low. Higher education level was significantly associated with greater awareness, highlighting a key demographic correlate. However, significant educational, financial, and infrastructural barriers hinder the transition from awareness to adoption. Policy reforms and structured training programs are urgently needed to support the integration of telerehabilitation into routine physiotherapy practice in Jordan.

## 1. Introduction

Telehealth has become an increasingly important component of modern healthcare systems and refers to the use of communication technologies such as video conferencing, mobile applications, and online platforms to deliver healthcare services remotely. It is considered a broad concept that includes several related terms such as telemedicine, telecare, and telerehabilitation. Telemedicine mainly focuses on remote medical consultations and clinical services, while telecare generally involves monitoring and supporting patients in their homes through digital [1,2]. Telerehabilitation specifically refers to the delivery of rehabilitation services such as physiotherapy assessment, treatment, and follow-up using telecommunication technologies. Over the past decade, telehealth services have expanded significantly due to the rapid development of digital technologies and the increasing need to improve access to healthcare services. The COVID-19 pandemic further accelerated the adoption of telehealth solutions worldwide, as healthcare systems relied on remote care to maintain healthcare delivery while minimizing physical contact between patients and healthcare providers [3]. Previous research has also highlighted that telehealth services can improve access to care for patients living in rural or underserved areas while reducing travel time and healthcare costs for both patients and providers [4].

Within physiotherapy practice, telerehabilitation has gained increasing attention as a potential method for delivering rehabilitation services remotely. Physiotherapists can use telerehabilitation to assess patients, supervise therapeutic exercises, monitor patient progress, and provide education without requiring patients to visit healthcare facilities physically. The emergence of advanced telepresence technologies, including high-definition video conferencing, haptic feedback systems, and wearable sensors has further expanded the potential for remote rehabilitation to approximate in-person care [5]. These technologies enable real-time interactive assessment and guidance that can be particularly valuable for conditions requiring hands-on supervision. Telerehabilitation can be applied across several physiotherapy specialties including musculoskeletal rehabilitation, neurological rehabilitation, cardiopulmonary physiotherapy, and pediatric rehabilitation [5]. In many cases, patients recovering from orthopedic surgeries or managing chronic musculoskeletal disorders may benefit from remote rehabilitation programs that allow continuous monitoring and guidance. Telerehabilitation services can be delivered through different communication models. Synchronous approaches involve real-time interaction between therapists and patients through video consultations, while asynchronous approaches allow patients to perform exercises independently and share recorded videos or progress reports for later review [6,7,8]. Hybrid rehabilitation models that combine traditional face-to-face treatment with remote follow-up sessions have also been suggested as an effective approach for maintaining continuity of physiotherapy care [9].

Globally, telerehabilitation has been increasingly recognized as an effective strategy to improve access to rehabilitation services and address growing healthcare demands. Many countries have integrated telehealth technologies into healthcare systems, particularly after the rapid digital transformation that occurred during the COVID-19 pandemic. However, the implementation of telerehabilitation varies considerably between healthcare systems and regions. Several facilitating factors may support the adoption of telerehabilitation, including improved technological infrastructure, better internet connectivity, and increased familiarity with digital technologies among healthcare professionals and patients [10]. At the same time, several barriers may limit its widespread implementation. These barriers include lack of training among healthcare providers, concerns regarding patient privacy and data security, and the absence of clear regulatory frameworks governing telehealth practices [4]. In addition, some healthcare professionals remain uncertain about the effectiveness of remote rehabilitation compared with traditional in-person physiotherapy treatment, which may also influence their willingness to adopt telehealth services in clinical practice [8,11].

In Jordan, the healthcare sector has gradually begun to explore the use of digital health technologies in recent years. Physiotherapy services are widely provided across hospitals, rehabilitation centers, and private clinics, and physiotherapists play an important role in managing various health conditions. Despite the growing international interest in telehealth services, the integration of telerehabilitation into routine physiotherapy practice in Jordan remains relatively limited compared with other countries. Several factors may influence the adoption of telehealth technologies within the healthcare system, including technological infrastructure, institutional support, and healthcare professionals’ willingness to adopt digital health tools [12]. In addition, the COVID-19 pandemic increased awareness of telemedicine and telehealth solutions worldwide, including in the Middle East region [12,13]. Nevertheless, the level of awareness and willingness among physiotherapists in Jordan to implement telerehabilitation services in their clinical practice remains an area that requires further investigation [14,15].

Several studies conducted internationally have examined the effectiveness and feasibility of telerehabilitation programs in physiotherapy practice. Research has demonstrated that telerehabilitation can produce clinical outcomes comparable to traditional face-to-face rehabilitation, particularly for musculoskeletal conditions and neurological disorders [16]. In addition, systematic reviews have reported that telerehabilitation may improve functional outcomes and patient engagement in rehabilitation programs [17]. During the COVID-19 pandemic, many physiotherapy services were delivered through telehealth platforms, and several studies reported high levels of patient satisfaction with remote physiotherapy services [18]. Patients often appreciate the convenience of receiving rehabilitation services at home and avoiding transportation barriers and long waiting times. Despite these promising findings, the successful implementation of telerehabilitation also depends on healthcare professionals’ attitudes and acceptance of telehealth technologies.

Although telerehabilitation has been widely discussed in international research, limited evidence is available regarding physiotherapists’ awareness and perceptions of telerehabilitation in Jordan. Understanding healthcare professionals’ attitudes toward telehealth technologies is essential for successful implementation of digital health services. Physiotherapists who lack knowledge or confidence in using telehealth platforms may be less likely to adopt these technologies in their clinical practice [18]. Therefore, educational initiatives, professional training programs, and clear clinical guidelines may help increase physiotherapists’ awareness and improve their readiness to integrate telerehabilitation into physiotherapy practice [19]. Identifying healthcare professionals’ perceptions and willingness can provide valuable insights for healthcare institutions and policymakers when developing strategies to support the implementation of telehealth services in rehabilitation care.

For the purposes of this study, we distinguish between several related but distinct concepts. Awareness refers to having knowledge about the existence and basic features of telerehabilitation. Use refers to the actual application of telerehabilitation in clinical practice, whether currently or in the past. Attitude encompasses the evaluative judgments and beliefs that physiotherapists hold about telerehabilitation, including perceived benefits and concerns. Willingness refers to the intention or inclination to adopt telerehabilitation in future practice. Finally, adoption refers to the integration of telerehabilitation into routine clinical practice. This study focuses primarily on awareness, use, attitudes, and perceived barriers, which collectively inform understanding of the factors that may facilitate or hinder adoption. Therefore, the aim of this study is to explore the awareness, perceptions, and willingness of physiotherapists in Jordan regarding the use of telerehabilitation in physiotherapy practice and to identify potential barriers and facilitators that may influence its adoption in clinical settings.

## 2. Materials and Methods

This study employed a quantitative cross-sectional design, which involved collecting data through an online self-administered questionnaire from participants between January and February 2026. Data collection occurred during a period when the Jordanian healthcare system was continuing to recover from the COVID-19 pandemic, with increased but still limited formal telehealth policies in place. This approach allows us to obtain a clear picture of the participants’ experiences and perceptions regarding telerehabilitation, providing valuable insights into their current knowledge and practices.

### 2.1. Participants

This study employed a convenience sampling strategy. Due to limited time and resources, participants were selected based on their accessibility and willingness to participate. This allowed identification of trends and barriers associated with telerehabilitation practice. A total of 250 physiotherapists were invited to participate in this study. Of these, 200 physiotherapists (111 males, 89 females; ages 23–62) completed and returned the questionnaire, yielding a response rate of 80%. The remaining 50 physiotherapists were excluded from the study due to either declining participation or failing to complete the questionnaire in full. We conducted an a priori sample size calculation using GPower software (version 3.1.9.7; Heinrich-Heine-Universität Düsseldorf, Düsseldorf, Germany) for a chi-square test of independence (family: Goodness-of-fit tests, Contingency tables). As several bivariate comparisons were planned (age, sex, education level, years of experience, working hours, and work setting, each against awareness), we calculated the sample size based on the most demanding of these variables; education level (4 categories) by awareness (2 categories), with degrees of freedom (df) = 3. Using a medium effect size (w = 0.3), a significance level of α = 0.05, and statistical power of 0.95, the required sample size was 191 participants. This approach ensured adequate power for all other planned comparisons with fewer categories. Thus, the 200 participants recruited in this study provided an adequate sample of Jordanian physiotherapists.

Participants in this study were required to have at least 1 year of experience as registered physiotherapists and to be currently engaged in clinical practice before completion of this online questionnaire. These criteria were verified through screening questions at the beginning of the questionnaire: participants were asked to report their years of experience and their current primary work setting. Those who reported less than 1 year of experience or who were not currently in clinical practice were automatically excluded and thanked for their interest. Physiotherapists working only in academic roles (full-time teaching without clinical practice) were not eligible for inclusion, as the study focused on clinical practice experiences. However, physiotherapists who combined academic and clinical roles were included if they met the clinical practice criterion. The study was conducted in accordance with the Declaration of Helsinki, and the protocol was approved by the Institutional Review Board at the Faculty of Applied Medical Sciences, Applied Science Private University (AMS-2025-6), ensuring that the research adhered to ethical guidelines and standards for studies involving human participants.

### 2.2. Assessments

The participating physiotherapists completed a self-administered online questionnaire adapted from a previously validated published questionnaire [8,20]. The adaptation process involved several specific modifications. First, we removed context related to the COVID-19 pandemic in the Libyan questionnaire [8] to ensure relevance beyond the pandemic era. Specifically, we removed references to ‘during the COVID-19 pandemic’ from the item wording and deleted three items that specifically referenced pandemic-related practice changes. Second, we added three new items based on a review of existing literature [4,11] focusing on payment policies (‘I am able to receive payment for telerehabilitation services’), technical difficulties (‘I have encountered technical difficulties when using telerehabilitation’), and infrastructure issues (‘Infrastructure issues are barriers to telerehabilitation adoption’). Third, the questionnaire was linguistically and culturally adapted for the Jordanian context: terminology was reviewed for clarity, and culturally appropriate phrasing was used while maintaining consistency with professional physiotherapy language in Jordan. The questionnaire was translated to ensure clarity for Arabic-speaking participants while retaining the original English version for reference. To ensure content validity of the newly added items and the overall adapted instrument, the questionnaire was reviewed by a panel of five physiotherapy academics and clinicians with expertise in telehealth and rehabilitation. Each panel member independently assessed the relevance, clarity, and comprehensiveness of all items using a 4-point content validity index (CVI) scale. Items with CVI scores below 0.78 were revised. Following expert review, the questionnaire was pilot-tested with 10 physiotherapists (5 male, 5 female; age range 25–55 years; not included in the main study sample) to assess clarity, comprehensibility, and completion time. Pilot participants were asked to complete the questionnaire and provide feedback on item wording, clarity, and relevance. Based on pilot feedback, minor wording adjustments were made to three items for improved clarity (e.g., simplifying technical terminology in the infrastructure question), and one item was rephrased to improve comprehension. The average completion time was 12 min, which was deemed acceptable. To assess the reliability of this questionnaire, a test–retest reliability analysis was conducted by administering the questionnaire twice to 25 physiotherapists with 14 days interval. The ICC was calculated using a two-way mixed-effects model, absolute agreement definition, and single measures type (ICC(2,1)). The overall instrument yielded an ICC of 0.81 (95% CI: 0.69–0.89), indicating good reliability. Section-level ICCs were also calculated: Section 1 (demographics) ICC = 0.89 (95% CI: 0.78–0.94), Section 2 (awareness and use) ICC = 0.78 (95% CI: 0.62–0.87), Section 3 (attitudes) ICC = 0.82 (95% CI: 0.70–0.90), and Section 4 (barriers) ICC = 0.79 (95% CI: 0.64–0.88). These reliability scores support the use of the questionnaire as a consistent and dependable instrument for our study. Online recruitment was used to invite physiotherapists to participate in our study. The questionnaire link was distributed via multiple channels, including online professional groups, public and private hospitals, private physiotherapy centers, and other settings with access to physiotherapists, such as universities. An information sheet was provided at the beginning of the online questionnaire, explaining the study’s purpose and emphasizing that participation was completely voluntary, anonymous, and confidential. Participants were explicitly informed of their right to withdraw from the study at any point before submission without consequence. Survey responses were collected using Google Forms with anonymity enabled (no IP addresses or identifying information were recorded). Data were stored on a password-protected institutional Google Drive account accessible only to the research team. To ensure confidentiality during workplace-based distribution, the questionnaire link was disseminated through general professional channels rather than through individual supervisors or administrators. No supervisors or administrators had access to information regarding which individuals participated or their responses. Also outlining the time commitment involved. The research team was available to provide further details for those who had additional questions, to support their understanding of the study. Informed consent was provided electronically before starting the questionnaire. Further, all participants were screened to confirm they met the inclusion criteria. This process ensured that all participants were fully informed and comfortable with contributing to our research.

**Figure 1:**
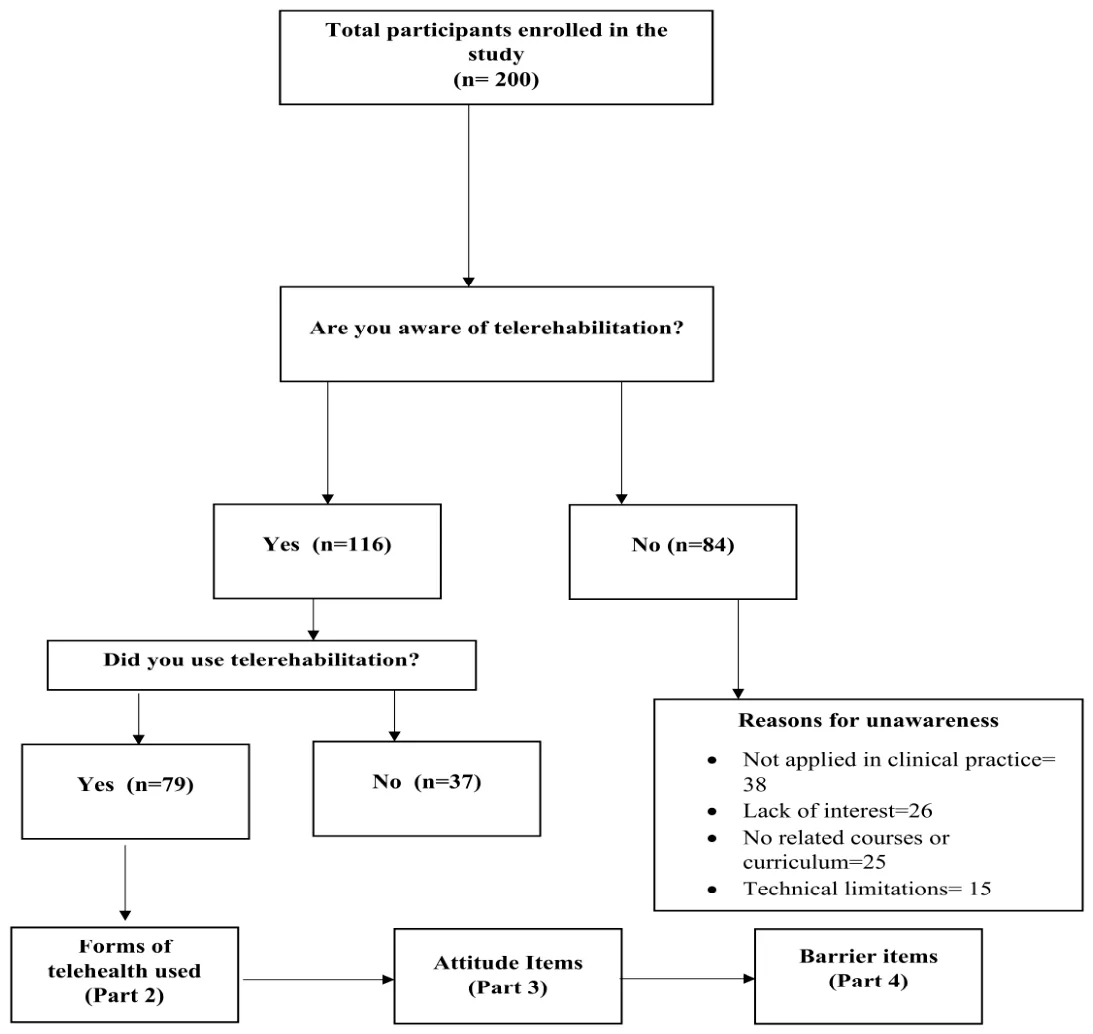
Flow diagram of questionnaire routing logic. Shaded boxes indicate items presented only to specific subgroups. Attitude and barrier items (Parts 3 and 4) were presented to all aware participants (n = 116), while detailed use items (Part 2) were presented only to those who reported actual use (n = 79).

The questionnaire collected personal information and evaluated their knowledge of telerehabilitation. The questionnaire is designed to be concise yet comprehensive, and it includes four major parts with a total of 32 items. The personal information section (Part 1, 8 items) included questions about age, sex, workplace, educational level, years of experience, and working hours. The second section (Part 2, 10 items) assessed awareness, use, and practice of telerehabilitation; it began with a contingency question to evaluate participants’ awareness of telerehabilitation and whether they used it or not, followed by questions about the different forms of telerehabilitation used and the most common app-based outcome measures, and additional questions about whether participants can use telerehabilitation in their current practice and whether they receive any payments for this service. The third section (Part 3, 9 items) contained questions about the perception and attitude of the physiotherapist about telerehabilitation, e.g., whether telerehabilitation enhances patient access and ensures confidentiality, whether telerehabilitation is acceptable to monitor outcomes and whether it allows more rapid monitoring. The final section (Part 4, 5 items) was related to the barriers and challenges encountered by the physiotherapists. The questionnaire items were carefully worded to maintain consistency with the definitions provided in the introduction. Specifically, ‘telerehabilitation’ was used in items referring to physiotherapy-specific remote rehabilitation services, while ‘telehealth’ was used in items referring to the broader concept of remote healthcare delivery that encompasses multiple disciplines. We reviewed all items to confirm that this distinction was maintained throughout the questionnaire. The questionnaire employed skip logic (branching) to ensure that participants only received items relevant to their experience. The routing logic was as follows: All participants received the initial awareness question: ‘Are you aware of telerehabilitation?’ Participants who answered ‘No’ to the awareness question were directed to a follow-up question about their reason for being unaware and then skipped all subsequent sections on use, practice, attitudes, and barriers. Participants who answered ‘Yes’ to the awareness question (n = 116) proceeded to the use question: ‘Did you use telerehabilitation?’ Participants who answered ‘Yes’ to the use question (n = 79) received detailed questions about forms of telehealth used and app-based outcome measures. All aware participants (n = 116) received the attitude and perception items (Part 3) and the barrier items (Part 4), regardless of whether they had personally used telerehabilitation, as these items assessed general perceptions and perceived barriers rather than hands-on experience. This design ensured that items presupposing hands-on use were only presented to participants who had actually used telerehabilitation, while general perception and barrier items were presented to all aware participants. The practice-related item (‘Is telerehabilitation used in your practice?’) and the payment-related item (‘Able to receive payment for telerehabilitation services?’) were presented to all participants who reported awareness of telerehabilitation (n = 116), regardless of whether they had personally used telerehabilitation. These items were designed to assess the broader practice context and payment environment relevant to aware physiotherapists, rather than individual usage experience. A flow diagram illustrating this routing logic is provided in Figure 1.

### 2.3. Data Synthesis and Statistical Analysis

The data analysis was carried out by one of the coauthors with expertise in statistical methods using SPSS Statistics (Version 23.0; IBM Corp., Armonk, NY, USA). The data were encoded and analyzed, with descriptive statistics computed using frequencies and percentages for the assessed variables.

In addition, to identify factors associated with awareness of telerehabilitation, inferential analyses were performed. A series of chi-square tests was executed to assess the bivariate associations between awareness of telerehabilitation and key categorical independent variables (age, sex, education level, years of experience, number of working hours, and work setting). Consistent with the exploratory aim of this study, these analyses were conducted as a descriptive, hypothesis-generating screen rather than confirmatory hypothesis tests. No adjustment for multiple comparisons was applied, as the primary pre-specified analysis was the association with education level, while the remaining analyses were considered hypothesis-generating. Results should be interpreted accordingly, with the primary finding being the education association. Before conducting the chi-square tests, we assessed the assumptions for Pearson’s chi-square test by examining expected cell counts. For analyses where more than 20% of cells had expected frequencies less than 5, we note this in the results and interpret findings with appropriate caution. For the education level analysis, 1 cell (12.5%) had an expected count less than 5 (minimum expected = 4.20), which marginally violated the assumption but was considered acceptable given the small violation and the robustness of chi-square to minor violations. For analyses with 2 × 2 tables (sex, working hours), Fisher’s exact test was reported alongside the chi-square results due to the small sample in some cells. For work setting, the original seven categories were collapsed into three clinically meaningful groups (hospital-based, private/community practice, other) to address expected count violations.

## 3. Results

### 3.1. Demographic and Professional Characteristics of Participants

A total of 200 Jordanian physiotherapists participated in this study. The demographic and professional characteristics of the sample are presented in Table 1. The majority of participants were aged 20–30 years (54.5%), male (55.5%), held a Bachelor’s degree (72.5%), and had less than 5 years of experience (45.0%). Most of the participants worked more than 8 h per day (63.0%).

### 3.2. Awareness and Use of Telerehabilitation

Of the 200 participants, 116 (58.0%) reported being aware of telerehabilitation, while 84 (42.0%) were not. Among those unaware, the primary reasons were ‘not applied in clinical practice’ (19.0%), ‘lack of interest’ (13.0%), and ‘no related courses or curriculum’ (12.5%). Among the aware participants, 79 (68.1% of aware, 39.5% of total sample) had used telerehabilitation at some point, with online video (27.0% of total sample; 46.6% of users) being the most common modality. Of those who had used telerehabilitation, 74 (93.7% of users, 37.0% of total sample) reported that telerehabilitation is currently used in their practice. A flowchart illustrating the relationship between these subgroups is provided in Figure 1. The most frequently used app-based outcome measures were range of motion (16.5%) and pain (15.0%). A majority (63.0%) reported that telerehabilitation is not used in their practice via live video conference, and 66.0% were unable to receive payment for telehealth services. Among aware participants who responded to attitude items, 51.0% of the total sample (87.9% of aware respondents) agreed that telerehabilitation is a valuable addition to practice, and 45.5% of the total sample (78.4% of aware respondents) expressed willingness to expand its use. All details are listed in Table 2.

### 3.3. Perceptions and Attitudes Towards Telerehabilitation

Participant perceptions are detailed in Table 3. Of those who responded, most held positive views on the benefits of telerehabilitation. However, a notable proportion (43.0%) believed it can never replace in-person consultation. Key barriers included technical difficulties (47.0% experienced frequently/sometimes), lack of training (34.0% Agree/Strongly Agree), and infrastructure issues (39.0% Agree/Strongly Agree).

### 3.4. Factors Associated with Awareness of Telerehabilitation

A chi-square test of independence revealed a statistically significant association between awareness of telerehabilitation and education level, χ^2^(3, N = 200) = 20.368, *p* < 0.001. Cramér’s V = 0.319, indicating a moderate association. Physiotherapists with higher educational degrees (Master’s and Ph.D.) demonstrated greater awareness than those with a Bachelor’s or Diploma (Table 4). No significant associations were found with age [χ^2^(4, N = 200) = 2.531, *p* = 0.639], sex [χ^2^(1, N = 200) = 0.570, *p* = 0.450], years of experience [χ^2^(4, N = 200) = 3.486, *p* = 0.480], working hours [χ^2^(1, N = 200) = 0.751, *p* = 0.386], or work setting [χ^2^(8, N = 200) = 6.320, *p* = 0.611]. Table 5 lists the complete details of all chi-square analyses. When the 84 unaware participants were excluded from analysis (leaving n = 116 aware participants), the association between education level and awareness of telerehabilitation remained statistically significant, χ^2^(3, n = 116) = 15.42, *p* = 0.001, confirming that the education effect was not driven by the presence of unaware participants.

## 4. Discussion

This cross-sectional study included 200 participants, aiming to investigate telerehabilitation awareness among Jordanian physiotherapists, the prevalence of telerehabilitation use, and opinions and attitudes towards telerehabilitation.

Our findings revealed a moderate level of awareness (58.0%) among Jordanian physiotherapists, which is comparable to developing countries contexts, where physiotherapists usually express positive attitudes toward telerehabilitation but demonstrate limited confidence, preparedness, or knowledge regarding the practical implementation [21,22]. Additionally, 39.5% have utilized telerehabilitation; this remains below the high levels of therapist uptake and population-level eligibility reported in Canadian and European contexts [23]. However, differences between countries should be interpreted cautiously, as variations in sampling, survey instruments, reimbursement systems, and the timing of data collection in relation to the pandemic may explain part of the difference. These inconsistent findings might be understood due to the robust digital health infrastructure in those countries, when compared to Jordan. It is important to note that the questionnaire used in this study was adapted from a previously published instrument [8] that shares authorship with the current manuscript. This relationship is disclosed to ensure transparency, and our adaptation involved substantial modifications for the Jordanian context and removal of pandemic-specific content.

Moreover, 42.0% of participants stated that they are unaware of telerehabilitation. This might be interpreted as a general professional preparation issue rather than mere individual lack of care. Among those unaware participants, 12.5% attributed this to the absence of telerehabilitation content in their courses, while 19.0% reported a lack of exposure or information, and 13.0% stated a low personal interest. Our results are comparable to previous findings in the literature, which reported that physiotherapy students and trainees frequently described missing curriculum content and limited exposure as the main reasons for not knowing about telerehabilitation, despite recognizing its growing relevance [24,25,26].

One of the major findings of this study is the significant association between higher education level and telerehabilitation awareness (*p* < 0.001). Our findings show that physiotherapists with master’s and Ph.D. degrees were more likely to be aware of telerehabilitation compared to those with a Bachelor’s degree or Diploma. Comparable findings were reported in previous investigations, which suggested that physiotherapists with higher degrees are more likely to adopt innovations in healthcare [27,28,29,30]. A previous study conducted in Austria and Germany found that higher educational level was a main predictor of higher readiness to implement telerehabilitation, stronger digital abilities, and positive affect toward its use [30]. However, we acknowledge that this is an association, not a causal relationship. The observed association may also reflect other factors that correlate with higher education, such as greater research exposure, more favorable institutional settings, or increased access to digital resources and professional networks. Furthermore, other investigations have suggested that postgraduate training and continued professional education significantly enhance digital skills and confidence, which often includes more exposure to emerging practices and professional development opportunities that may not be available at the undergraduate level [27,28,29]. Therefore, while higher education is associated with awareness, we cannot conclude that it directly causes greater adoption or use of telerehabilitation.

Furthermore, previous findings suggest that digital health is still under-represented in basic physiotherapy competency standards; additionally, it is considered an elective or advanced capability, rather than a core skill for all graduates [27,31]. This was also reported in recent scoping and systematic reviews, highlighting major gaps in digital health content at the undergraduate level and calling for its systematic integration across curricula [28,32,33]. Among 45.5% of our participants who were aware of telerehabilitation and showed willingness to use it, there is still a systemic barrier that prevents this clinical transition.

One of the major barriers was the reimbursement deficit, which could be noticed when 33.0% of the total sample (56.9% of aware participants) reported they could not receive payment for telerehabilitation services. The questionnaire item asked specifically: ‘Are you able to receive payment for telerehabilitation services?’ without distinguishing between different payment mechanisms. Based on participant comments and the Jordanian context, this likely encompasses multiple situations: lack of formal insurance reimbursement policies, inability to charge patients directly for remote services, and absence of institutional payment mechanisms for telehealth. This is a crucial policy challenge that renders telerehabilitation financially and makes it financially unsustainable for many health care providers, particularly in private practice or resource-limited settings [34,35,36,37].

Our findings also reveal that Jordanian physiotherapists preferred online video (27.0%) and smartphone-based communication (13.0%) rather than specialized rehabilitation platforms. Moreover, these tools were mainly utilized by physiotherapists to assess Range of Motion (16.5%) and Pain (15.0%). These patterns demonstrate heavy reliance on low-tech video and smartphone tools in addition to focusing only on a narrow set of outcomes, such as ROM and pain scales, which indicates a developing stage of telerehabilitation maturity. In other words, basic clinical monitoring is used, but more comprehensive, digitally integrated assessments, such as performance-based tests and complex functional scales, remain underused [5,17,38,39]. This might be interpreted by the disparities in digital infrastructure, the lack of dedicated telerehabilitation platforms, and appropriate training [17,38,40,41,42].

Another interesting finding of this study is the physiotherapists’ workload, 63.0% of participants work more than 8 h per day. While our study did not directly measure burnout or technostress, previous research suggests that long working hours may be closely linked to burnout, technostress, and resistance to change among healthcare professionals, which was reported previously in the literature [40,42,43]. A recent umbrella review by S. J. Oudbier et al. suggested that concerns about increased working hours or workload are a common barrier to using digital technologies [40]. Another important factor is that healthcare practitioners might perceive electronic health records as sources of daily frustration and burnout, with higher after-hours documentation and message load strongly associated with exhaustion and emotional fatigue [44]. However, these are potential explanations that warrant further investigation rather than conclusions directly supported by our data.

### Strengths and Limitations

This study has several strengths: firstly, this study provides a snapshot of the Jordanian physiotherapy workforce, spanning private, public, and university settings. Additionally, the sample size (N = 200) provides a robust cross-section for a middle-income country context. However, we acknowledge that the convenience sampling method means our findings may not be fully representative of all physiotherapists in Jordan. Some professional settings may be overrepresented (e.g., those with access to online professional networks) or underrepresented (e.g., physiotherapists in rural areas or those with limited internet access). Finally, this study provided a comprehensive analysis of multiple factors that contribute to telerehabilitation awareness and attitude, including financial barriers, technical difficulties, education and infrastructural issues. However, the study is not without limitations. First, the 42.0% unawareness rate and the resulting skip logic meant that 84 participants did not receive attitude and barrier items. This represents a structural missing-not-at-random (MNAR) problem, rather than ordinary missing data, because the absence of responses was systematically determined by participants’ awareness status. This limits interpretation of the overall percentages and means that attitude findings reflect the views of only the aware subsample. The true population-level attitudes toward telerehabilitation may differ if unaware participants were to become aware. Second, the study relied on self-reported data, which may be subject to recall bias, social desirability bias, and overestimation of positive attitudes or behaviors. Third, online recruitment may have introduced selection bias, as physiotherapists with greater digital literacy or interest in technology may have been more likely to participate. Fourth, the convenience sampling method and lack of a probability sample mean that the findings may not be representative of all Jordanian physiotherapists. Fifth, the higher percentage of younger professionals aged 20–30 (54.5%) may overrepresent the ‘digital native’ perspective. Sixth, non-response bias cannot be ruled out, as 20% of invited physiotherapists either declined or failed to complete the questionnaire, potentially because they were not interested in digital technology or were busy with heavy clinical workloads. It is possible that those non-respondents differ completely from respondents. Future studies should aim for higher response rates and consider probability sampling methods and address the MNAR problem through strategies such as awareness-raising interventions before surveying.

This study was conducted in Jordan, a middle-income country with a developing digital health infrastructure. Based on the contextual similarities in healthcare systems, educational structures, and digital infrastructure development, our findings may be qualitatively transferable to countries with similar characteristics, including some Middle Eastern countries, North African countries, and certain Asian countries where telerehabilitation remains in early adoption stages. However, we acknowledge that this transferability inference is based on qualitative contextual similarities rather than direct comparative data, and should be interpreted with appropriate caution. Similarity in income level alone does not ensure similarity in health policy, digital infrastructure, or professional regulation. Additionally, variations in professional regulation, cultural attitudes toward remote care, and the status of physiotherapy as a profession may influence transferability. Our findings are less likely to be transferable to high-income countries, countries with very established digital infrastructure, or countries that have integrated telerehabilitation training in physiotherapy curriculum.

## 5. Conclusions

The results of this study suggest that education level is significantly associated with telerehabilitation awareness among Jordanian physiotherapists. The transformation from awareness to clinical adoption is hindered by multiple perceived barriers including financial reimbursement, technical difficulties, lack of training, and infrastructure issues. These findings highlight the need for policy reforms addressing reimbursement mechanisms, alongside structured training programs to support the integration of telerehabilitation into routine physiotherapy practice in Jordan.

## Figures and Tables

**Table 1:** Demographic and professional characteristics of participants.

Characteristic	Category	Frequency (n)	Percent
Age (years)	20–30	109	54.5
	31–40	48	24.0
	41–50	24	12.0
	51–60	14	7.0
	≥60	5	2.5
Sex	Female	89	44.5
	Male	111	55.5
Education Level	Bachelor	145	72.5
	Diploma	14	7.0
	Master	31	15.5
	Ph.D.	10	5.0
Experience (years)	<5	90	45.0
	5–10	44	22.0
	11–15	33	16.5
	16–20	21	10.5
	>20	12	6.0
Working hours/day	<8	74	37.0
	≥8	126	63.0
Work Setting	Private Practice	71	35.5
	Public Hospital	66	33.0
	Private Hospital	44	22.0
	Private center	9	4.5
	Gym	4	2.0
	Others	3	1.5
	University	3	1.5

**Table 2:** Awareness, use, and practice of telerehabilitation.

Question	Category	n	% of Total (n = 200)	% of Eligible
Are you aware of telerehabilitation?	Yes **	116	58.0%	-
	No *	84	42.0%	-
If NO, what is the main reason? *	Not applied in clinical practice	38	19.0%	45.2%
	Lack of interest	26	13.0%	31.0%
	No related courses or curriculum	25	12.5%	29.8%
	Technical limitations	15	7.5%	17.9%
	Other (specified)	8	4.0%	9.5%
	No response	-	-	-
Did you use telerehabilitation? **	Yes ***	79	39.5%	68.1%
	No	37	18.5%	31.9%
What forms of telehealth have you used? ***	Online video	54	27.0%	46.6%
	Smartphone	26	13.0%	22.4%
	Recorded video or messages	21	10.5%	18.1%
	Telephone	11	5.5%	9.5%
	E-mail	4	2.0%	3.4%
Most common app-based outcome measures ***	Range of motion	33	16.5%	28.4%
	Pain	30	15.0%	25.9%
	Gait	14	7.0%	12.1%
	Muscle strength	13	6.5%	11.2%
	Other	26	13.0%	22.4%
Is telerehabilitation used in your practice? **	Yes	74	37.0%	63.8%
	No	42	21.0%	36.2%
Able to receive payment for telerehabilitation services? **	No	66	33.0%	56.9%
	Yes, since COVID-19	42	21.0%	36.2%
	Yes, since before COVID-19	8	4.0%	6.9%
Telerehabilitation is a valuable addition **	Agree	102	51.0%	87.9%
	Neutral	10	5.0%	8.6%
	Disagree	4	2.0%	3.4%
Willing to expand use in the future **	Agree/Strongly Agree	91	45.5%	78.4%
	Neutral	16	8.0%	13.8%
	Disagree/Strongly Disagree	9	4.5%	7.8%

For multiple-response (“select all that apply”) items, percentages may sum to more than 100%. * = Unaware participants (answered “No” to awareness; n = 84); ** = Aware participants (answered “Yes” to awareness; n = 116); *** = Participants who had used telerehabilitation (answered "Yes" to use among aware participants; n = 79).

**Table 3:** Perceptions and attitudes towards telerehabilitation.

Statement	Response	n (Eligible N = 116)	% of Total (N = 200)	% of Eligible
Telerehabilitation enhances patient access	Agree	85	42.5%	73.3%
	Neutral	22	11.0%	19.0%
	Disagree	9	4.5%	7.8%
Telehealth ensures confidentiality	Agree	84	42.0%	72.4%
	Neutral	24	12.0%	20.7%
	Disagree	8	4.0%	6.9%
Telehealth is acceptable to monitor outcomes	Agree	81	40.5%	69.8%
	Neutral	26	13.0%	22.4%
	Disagree	9	4.5%	7.8%
Telehealth allows more rapid monitoring	Agree	82	41.0%	70.7%
	Neutral	22	11.0%	19.0%
	Disagree	12	6.0%	10.3%
Confident in delivering treatment remotely	Agree	76	38.0%	65.5%
	Neutral	28	14.0%	24.1%
	Disagree	12	6.0%	10.3%
Telehealth is evidence-based	Agree	73	36.5%	62.9%
	Neutral	33	16.5%	28.4%
	Disagree	10	5.0%	8.6%
Telehealth is beneficial for providers	Agree	85	42.5%	73.3%
	Neutral	21	10.5%	18.1%
	Disagree	10	5.0%	8.6%
Security is important for implementation	Agree	87	43.5%	75.0%
	Neutral	19	9.5%	16.4%
	Disagree	10	5.0%	8.6%
Telerehabilitation cannot replace in-person consultation	Yes	86	43.0%	74.1%
	No, I could be more productive	30	15.0%	25.9%
Encountered technical difficulties	Frequently/Sometimes	94	47.0%	81.0%
	Rarely/Never	22	11.0%	19.0%
Lack of training limits my ability	Agree	68	34.0%	58.6%
	Neutral	34	17.0%	29.3%
	Disagree	14	7.0%	12.1%
Infrastructure issues are barriers	Agree	78	39.0%	67.2%
	Neutral	28	14.0%	24.1%
	Disagree	10	5.0%	8.6%

All items in this table were presented only to participants who reported awareness of telerehabilitation (n = 116). Percentages in the “% of total” column are calculated based on the total sample (N = 200). Percentages in the “% of eligible” column are calculated based on the eligible subgroup (n = 116).

**Table 4:** Association between education level and awareness of telerehabilitation.

Education Level	Aware of Telerehabilitation (n = 116)	Not Aware of Telerehabilitation (n = 84)	Total (N = 200)	*p*-Value
Bachelor	71 (61.2%)	74 (88.1%)	145 (72.5%)	
Diploma	9 (7.8%)	5 (6.0%)	14 (7.0%)	<0.001 *
Master	28 (24.1%)	3 (3.6%)	31 (15.5%)	
Ph.D.	8 (6.9%)	2 (2.4%)	10 (5.0%)	
Total	116 (100%)	84 (100%)	200 (100%)	

Data presented as n (%). *p*-value calculated using the Pearson Chi-Square test. * Statistically significant at *p* < 0.05.

**Table 5:** Association between age, sex, years of experience, working hours, and work setting and awareness of telerehabilitation.

Variable	χ^2^	df	*p*-Value
Age (years)	2.531	4	0.639
Sex	0.570	1	0.450
Years of Experience	3.486	4	0.480
Working Hours (hours/day)	0.751	1	0.386
Work Setting	6.320	8	0.611

Expected cell count diagnostics: education (1 of 8 cells, 12.5%, min = 4.20); age (2 of 10 cells, 20.0%, min = 2.10); sex (0 cells, min = 37.38); experience (0 cells, min = 5.04); working hours (0 cells, min = 31.08); work setting (original: 12 of 18 cells, 66.7%, min = 0.42; after collapse: 1 of 6 cells, 16.7%, min = 4.20). Fisher’s exact test was reported for 2 × 2 tables (sex, working hours).

## Data Availability

The data presented in this study are available on request from the corresponding author. Due to the sensitive nature of the data collected and to protect participant confidentiality, the data are not publicly available. However, de-identified data may be made available to qualified researchers upon reasonable request and subject to appropriate data sharing agreements to ensure confidentiality and ethical use.

## References

[1] World Health Organization (2017). Telemedicine: Opportunities and Developments in Member States.

[2] Bashshur R., Shannon G., Krupinski E., Grigsby J. (2011). The taxonomy of telemedicine. Telemed. J. E-Health.

[3] Dorsey E.R., Topol E.J. (2016). State of telehealth. N. Engl. J. Med..

[4] Kruse C.S., Krowski N., Rodriguez B., Tran L., Vela J., Brooks M. (2017). Telehealth and patient satisfaction: A systematic review and narrative analysis. BMJ Open.

[5] Lee A.C., Harada N. (2012). Telehealth as a means of health care delivery for physical therapist practice. Phys. Ther..

[6] Russell T.G. (2007). Physical rehabilitation using telemedicine. J. Telemed. Telecare.

[7] Kairy D., Tousignant M., Leclerc N., Côté A., Levasseur M. (2013). The patient’s perspective of in-home telerehabilitation physiotherapy services following total knee arthroplasty. Int. J. Environ. Res. Public Health.

[8] Elmahgoub S.E.T., Ben Said A., Abu Khadra F., Aburub A., Tóth Á.L. (2025). The use of telerehabilitation among Libyan physiotherapists during the COVID-19 pandemic: A cross-sectional study. Int. J. Environ. Res. Public Health.

[9] Laver K., Adey-Ward M., Crotty M., Lannin N., George S., Sherrington C. (2020). Telerehabilitation services for stroke. Cochrane Database Syst. Rev..

[10] Tenforde A.S., Hefner J., Kodish-Wachs J., Iaccarino M., Paganoni S. (2017). Telehealth in physical medicine and rehabilitation: A narrative review. PM R.

[11] Scott Kruse C., Fohn J., Wilson N., Nunez-Patlan E., Zipp S., Mileski M. (2020). Utilization barriers and medical outcomes commensurate with the use of telehealth among older adults: Systematic review. JMIR Med. Inform..

[12] Al-Samarraie H., Ghazal S., Alzahrani A., Moody L. (2020). Telemedicine in Middle Eastern countries: Progress, challenges and opportunities. Int. J. Med. Inform..

[13] Upadhyaya G.K., Iyengar K., Jain V.K., Vaishya R. (2020). Challenges and strategies in management of osteoporosis and fragility fracture care during COVID-19 pandemic. J. Orthop..

[14] Alarabyat I.A., Al-Nsair N., Alrimawi I., Al-Yateem N., Shudifat R.M., Saifan A.R. (2023). Perceived barriers to effective use of telehealth in managing the care of patients with cardiovascular diseases: A qualitative study exploring healthcare professionals’ views in Jordan. BMC Health Serv. Res..

[15] Al-Dmour H., Masa’deh R., Salman A., Abuhashesh M., Al-Dmour R. (2020). Influence of social media platforms on public health protection against the COVID-19 pandemic via the mediating effects of public health awareness and behavioral changes: Integrated model. J. Med. Internet Res..

[16] Cottrell M.A., Galea O.A., O’Leary S.P., Hill A., Russell T.G. (2017). Real-time telerehabilitation for the treatment of musculoskeletal conditions: A systematic review and meta-analysis. Clin. Rehabil..

[17] Seron P., Oliveros M.J., Gutierrez-Arias R., Fuentes-Aspe R., Torres-Castro R.C., Merino-Osorio C., Nahuelhual P., Inostroza J., Jalil Y., Solano R. (2021). Effectiveness of telerehabilitation in physical therapy: A rapid overview. Phys. Ther..

[18] Tenforde A.S., Borgstrom H., Polich G., Steere H., Davis I.S., Cotton K., O’Donnell M., Silver J.K. (2020). Outpatient physical, occupational, and speech therapy synchronous telemedicine: A survey study of patient satisfaction with virtual visits during the COVID-19 pandemic. Am. J. Phys. Med. Rehabil..

[19] Cottrell M.A., Russell T.G. (2020). Telehealth for musculoskeletal physiotherapy. Musculoskelet. Sci. Pract..

[20] Gomes C.M., Favorito L.A., Henriques J.V.T., Canalini A.F., Anzolch K.M., Fernandes R.C., Bellucci C.H.S., Silva C.S., Wroclawski M.L., Pompeo A.C.L. (2020). Impact of COVID-19 on clinical practice, income, health and lifestyle behavior of Brazilian urologists. Int. Braz. J. Urol..

[21] Alrushud A., Alrawaili S., Alharthi A., Shaheen A., Alotaibi N., AlSabhan R., Alharbi S., Ali N., Mohammed E., Sweeh J. (2022). Physical therapists’ perceptions of and satisfaction with delivering telerehabilitation sessions to patients with knee osteoarthritis during the COVID-19 pandemic: Preliminary study. Musculoskelet. Care.

[22] Fernandes L.G., Oliveira R.R., Barros P.M., Fagundes F.R.C., Soares R.J., Saragiotto B.T. (2022). Physical therapists and public perceptions of telerehabilitation: An online open survey on acceptability, preferences, and needs. Braz. J. Phys. Ther..

[23] Giesbrecht E., Mulder M., Fricke M., Wener P., van Egmond M., Aarden J.J., Brown C.L., Pol M., van der Schaaf M. (2023). Telerehabilitation delivery in Canada and the Netherlands: Results of a survey study. JMIR Rehabil. Assist. Technol..

[24] Baroni M.P., Jacob M.F.A., Rios W.R., Fandim J.V., Fernandes L.G., Chaves P.I., Fioratti I., Saragiotto B.T. (2023). The state of the art in telerehabilitation for musculoskeletal conditions. Arch. Physiother..

[25] Ramalingam V., Thirumalai J., Wong L.S., Vasanthi R.K., Purushothaman V.K., Suganthirababu P., Annadurai B. (2025). Exploring telerehabilitation awareness, application, and future outlook in sports rehabilitation among physiotherapy students: A web-based survey. PeerJ.

[26] Başer Seçer M., Çırak T.Ö. (2022). Examination of telerehabilitation knowledge, awareness, and opinions of physical therapy and rehabilitation students. Med. Sci. Educ..

[27] Longhini J., Rossettini G., Palese A. (2022). Digital health competencies among health care professionals: Systematic review. J. Med. Internet Res..

[28] Kulju E., Jokinen E., Oikarinen A., Hammarén M., Kanste O., Mikkonen K. (2024). Educational interventions and their effects on healthcare professionals’ digital competence development: A systematic review. Int. J. Med. Inform..

[29] Bonet-Collantes M., Niño-Pinzón D.M., Chaustre-Porras A.D., Salas-Poloche Y.A., Angarita-Fonseca A. (2024). Enhancing physiotherapists’ knowledge and perceptions of telerehabilitation: A before-after educational intervention study. Physiother. Res. Int..

[30] Seebacher B., Bohrmann E., Geimer C., Kahraman T., Reindl M., Diermayr G. (2024). Factors influencing the willingness to adopt telerehabilitation among rehabilitation professionals in Austria and Germany: A survey comparing data before and during COVID-19. Disabil. Rehabil..

[31] Merolli M., Ahmed O., McCreesh K., Remedios L., Butler-Henderson K. (2024). Are physiotherapists expected to be competent in digital health practice? meta-synthesis of international physiotherapy practice competency standards. Physiother. Theory Pr..

[32] Kleib M., Arries A., Nagle L.M., Ali S., Idrees S., Costa D.D., Kennedy M., Darko E.M. (2024). Digital health education and training for undergraduate and graduate nursing students: Scoping review. JMIR Nurs..

[33] Aydınlar A., Mert A., Kütükçü E., Kırımlı E.E., Alış D., Akın A., Altıntaş L. (2024). Awareness and level of digital literacy among students receiving health-based education. BMC Med. Educ..

[34] Saravanakumar S., Oommen A. (2024). Evaluation of telehealth services that are clinically appropriate for reimbursement in the US Medicaid population: Mixed methods study. J. Med. Internet Res..

[35] Tuckson R.V., Edmunds M., Hodgkins M.L. (2017). Telehealth. N. Engl. J. Med..

[36] Porteny T., Brophy S.A., Burroughs E. (2025). Experiences of telehealth reimbursement policies in federally qualified health centers. JAMA Netw. Open.

[37] Li Q., Chen F., Zeng H., Xu J. (2024). Health insurance payment for telehealth services: Scoping review and narrative synthesis. J. Med. Internet Res..

[38] Stephenson A., Howes S., Murphy P.J., Deutsch J.E., Stokes M., Pedlow K., McDonough S.M. (2022). Factors influencing the delivery of telerehabilitation for stroke: A systematic review. PLoS ONE.

[39] Alwadai B., Lazem H., Almoajil H., Hall A.J., Mansoubi M., Dawes H. (2024). Telerehabilitation and Its Impact Following Stroke: An Umbrella Review of Systematic Reviews. J. Clin. Med..

[40] Oudbier S.J., Souget-Ruff S.P., Chen B.S.J., A Ziesemer K., Meij H.J., A Smets E.M. (2024). Implementation barriers and facilitators of remote monitoring, remote consultation and digital care platforms through the eyes of healthcare professionals: A review of reviews. BMJ Open.

[41] Lee A.Y.L., Wong A.K., Hung T.T.M., Yan J., Yang S. (2022). Nurse-led telehealth intervention for rehabilitation (telerehabilitation) among community-dwelling patients with chronic diseases: Systematic review and meta-analysis. J. Med. Internet Res..

[42] Borges do Nascimento I.J., Abdulazeem H., Vasanthan L.T., Martinez E.Z., Zucoloto M.L., Østengaard L., Azzopardi-Muscat N., Zapata T., Novillo-Ortiz D. (2023). Barriers and facilitators to utilizing digital health technologies by healthcare professionals. npj Digit. Med..

[43] Hasebrook J.P., Michalak L., Kohnen D., Metelmann B., Metelmann C., Brinkrolf P., Flessa S., Hahnenkamp K. (2023). Digital transition in rural emergency medicine: Impact of job satisfaction and workload on communication and technology acceptance. PLoS ONE.

[44] Li C., Parpia C., Sriharan A., Keefe D.T. (2022). Electronic medical record-related burnout in healthcare providers: A scoping review of outcomes and interventions. BMJ Open.

